# Evaluation of top-down mass spectral identification with homologous protein sequences

**DOI:** 10.1186/s12859-018-2462-1

**Published:** 2018-12-28

**Authors:** Ziwei Li, Bo He, Qiang Kou, Zhe Wang, Si Wu, Yunlong Liu, Weixing Feng, Xiaowen Liu

**Affiliations:** 1 College of Automation, Harbin Engineering University, 145, Nan Tong Street, Harbin, Heilongjiang, 150001 China; 2 Department of Medical and Molecular Genetics, Indiana University School of Medicine, 410 West 10th Street, Indianapolis, IN, 46202 USA; 3 Department of BioHealth Informatics, Indiana University-Purdue University Indianapolis, 719 Indiana Avenue, Indianapolis, IN, 46202 USA; 4 Department of Chemistry and Biochemistry, University of Oklahoma, 101 Stephenson Parkway, Norman, OK, 73019 USA; 5 Center for Computational Biology and Bioinformatics, Indiana University School of Medicine, 410 West 10th Street, Indianapolis, IN, 46202 USA

**Keywords:** Mass spectrometry, Top-down, Homologous protein database

## Abstract

**Background:**

Top-down mass spectrometry has unique advantages in identifying proteoforms with multiple post-translational modifications and/or unknown alterations. Most software tools in this area search top-down mass spectra against a protein sequence database for proteoform identification. When the species studied in a mass spectrometry experiment lacks its proteome sequence database, a homologous protein sequence database can be used for proteoform identification. The accuracy of homologous protein sequences affects the sensitivity of proteoform identification and the accuracy of mass shift localization.

**Results:**

We tested TopPIC, a commonly used software tool for top-down mass spectral identification, on a top-down mass spectrometry data set of *Escherichia coli* K12 MG1655, and evaluated its performance using an *Escherichia coli* K12 MG1655 proteome database and a homologous protein database. The number of identified spectra with the homologous database was about half of that with the *Escherichia coli* K12 MG1655 database. We also tested TopPIC on a top-down mass spectrometry data set of human MCF-7 cells and obtained similar results.

**Conclusions:**

Experimental results demonstrated that TopPIC is capable of identifying many proteoform spectrum matches and localizing unknown alterations using homologous protein sequences containing no more than 2 mutations.

**Electronic supplementary material:**

The online version of this article (10.1186/s12859-018-2462-1) contains supplementary material, which is available to authorized users.

## Background

Top-down mass spectrometry (MS) has become a widely-used technology for proteoform identification because it has unique advantages in analyzing modified proteoforms [1]. In the past two decades, the dominant technology in proteomics studies is bottom-up MS, in which long proteins are proteolytically digested in sample preparation, generating short peptides that are easy to be ionized and measured in mass spectrometers [2–4]. Compared with bottom-up MS, top-down MS skips protein digestion and directly analyzes intact proteins, making it suitable for identifying and characterizing proteoforms with post-translational modifications (PTM) in complex mixtures.

Database search is routinely used for spectral identification by top-down tandem mass spectrometry (MS/MS). In this approach, experimental MS/MS spectra are searched against theoretical spectra generated from database protein sequences to find high scoring proteoform spectrum matches (PrSMs). Many studies [5–7] have been carried out to design similarity scoring functions of PrSMs, improve the sensitivity of proteoform identification, and estimate statistical significance and false discovery rates of identifications. Various software tools have been developed for proteoform identification, such as ProSightPC [8], MS-Align+ [9], SpectroGene [10], TopPIC [11], TopMG [12], pTop [13], and MSPathFinder [14].

**Fig. 1:**
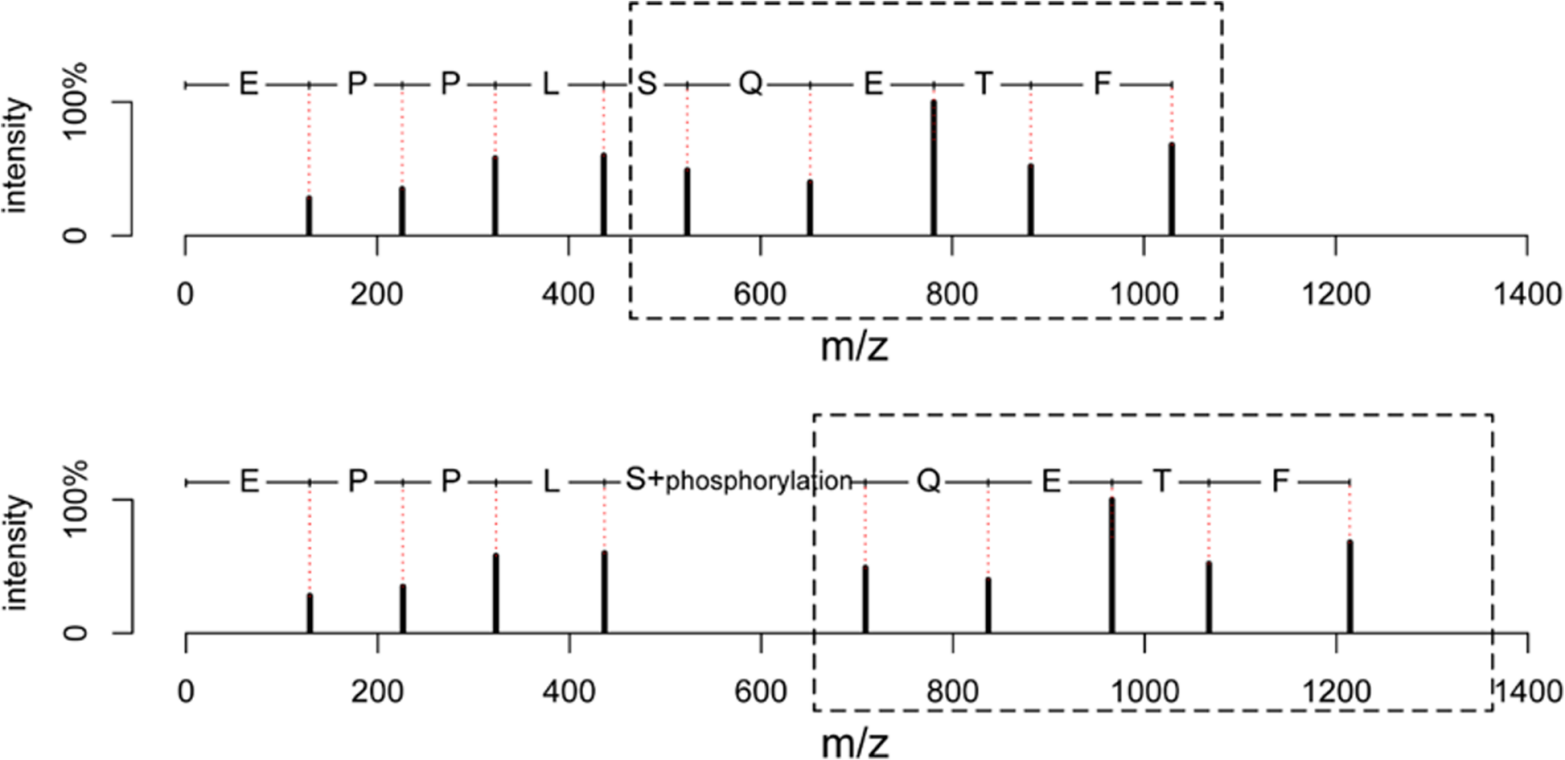
Comparison of the theoretical spectrum (top) of an unmodified protein sequence EPPLSQETFS and the theoretical spectrum (bottom) of a modified proteoform EPPLS[phosphorylation]QETFS, in which the serine residue is phosphorylated. Only N-terminal fragment peaks are included in the theoretical spectra to simply the comparison. The fragment peaks in the box are shifted to the right by 79.97 Da in the bottom spectrum because of the phosphorylation

When top-down MS/MS is used to analyze a species whose proteome database is unavailable, a homologous proteome database can be searched against for spectral identification. A homologous sequence contains mutations compared with the corresponding sequence of the species being studied. Alterations, such as PTMs and mutations, in a proteoform introduce mass shifts to peaks in its MS/MS spectra (Fig. 1). A low similarity score is often reported when we compare an experimental spectrum of a proteoform and a theoretical spectrum of a homologous database protein. As a result, a top-down MS/MS spectrum is elusive to identify by database search if the proteoform that produced it contains many alterations compared with the database sequence.

TopPIC is a commonly used software tool for the identification and characterization of proteoforms with unknown alterations by top-down MS/MS. In this paper, we present a method for proteoform identification by top-down MS using homologous protein sequences when the species being studied lacks a proteome database. We also study how mutations in homologous database protein sequences affect the performance of TopPIC on proteoform identification. Experimental results on a top-down MS/MS data set from *Escherichia coli* K12 MG1655 showed that TopPIC achieved high sensitivity in proteoform identification and high accuracy in mass shift localization when homologous protein sequences contain no more than 2 mutations.

## Methods

### Data sets

Two top-down MS/MS data sets were used to evaluate the performance of TopPIC and how mutations in database protein sequences affect the sensitivity and accuracy of proteoform identification: the first was from *Escherichia coli* (EC) and the second was from MCF-7 cells.

The EC data set was published in [11]. EC K12 MG1655 was grown in M9 minimal medium at 37 ^∘^C with shaking. Cells were harvested at 4 ^∘^C. Cell pellets were lysed by using 0.1 mM zirconia/silica beads. Cell debris and beads were removed by centrifugation, and the clarified cell lysate was subject to ultracentrifugation at 4 ^∘^C for sub-cellular fractionation. EC protein was separated by reverse phase liquid chromatography (RPLC) on a Waters NanoAquity system with a custom packed C5 column and analyzed by an LTQ Orbitrap Velos mass spectrometer. Parent spectra were collected at a 60,000 resolution and the top 4 ions in each MS spectrum were selected for MS/MS analysis, in which the resolution was 60,000 and the alternating fragmentation mode was used. A total of 2027 collisional-induced dissociation (CID) and 2027 electron-transfer dissociation (ETD) MS/MS spectra were generated. The raw file of the EC data was converted into an mzXML file using msconvert in ProteoWizard (version 2.0) [15], and the mzXML file was deconvoluted using MS-Deconv (version 0.8.0) [16] with default parameter settings.

The soluble MCF-7 intact proteins were separated using the bead-beating based cell lysis approach followed by a filter-based desalting step. A home-made long column was directly coupled on an LTQ Orbitrap Velos Pro mass spectrometer with a customized ion source. The MS/MS data were collected with data dependent CID and a resolution of 60,000. A total of 5310 MS/MS spectra were collected. The raw file of the MCF-7 data was converted into an mzML file using msconvert, and the mzML file was deconvoluted using TopFD in TopPIC suite [17] with default parameter settings.

### Protein databases

A proteome database of EC K12 MG1655 and a proteome database of EC ISC11 were used in the EC data analysis. The two proteome databases were downloaded from the UniProt database (version May 2016) [18]. The two databases are referred to as the K12 and ISC11 databases, respectively. There are 4314 entries in the K12 database and 6130 entries in the ISC11 database. A human proteome database and a mouse proteome database were downloaded from the UniProt database (version May 2018) for the analysis of the MCF-7 data set. The human database and the mouse database contain 20,328 and 16,966 proteins, respectively.

### Experiment design

**Fig. 2:**
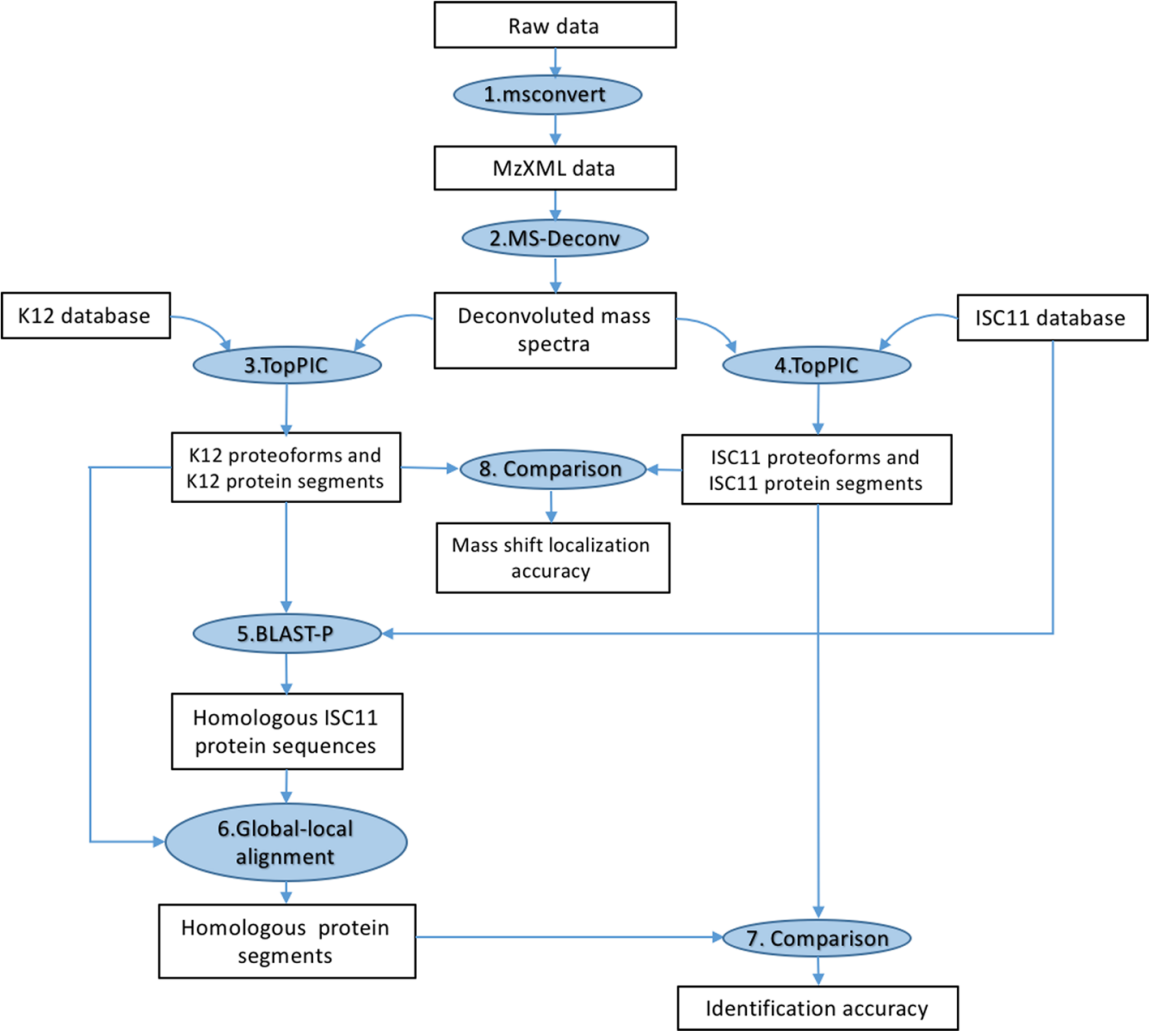
Outline of the experimental design. Raw MS data are converted to deconvoluted mass spectra, which are further searched against the K12 and ISC11 proteome databases separately. A K12 protein segment is obtained from each K12 proteoform identified from the K12 proteome database and searched against the ISC11 proteome database to find the best homologous ISC11 protein by BLAST-P. Then a global-local alignment between the homologous ISC11 protein sequence and the K12 protein segment is used to find the best-scoring homologous protein segment. Finally, homologous protein segments and ISC11 protein segments identified from the ISC11 proteome database are compared to evaluate the accuracy of the ISC11 protein segments, and ISC11 proteoforms are compared with K12 proteoforms to evaluate the accuracy of mass shift localization

The design of the EC data analysis is shown in Fig. 2. We tested two scenarios in proteoform identification: (1) a reference protein database is available and (2) no reference databases are available, but a homologous protein database is available. The K12 and ISC11 databases were used to evaluate the performance of TopPIC (version 1.1) in the two scenarios. We searched the deconvoluted spectra against the two protein databases separately for spectral identification.

### Proteoform identification using TopPIC

In TopPIC, a shuffled decoy database was concatenated to the target database, a 1% spectrum-level false discovery rate (FDR) was used for filtering identifications. The error tolerances for precursor and fragment masses were set to 15 parts-per-million (ppm), no fixed modifications were used, and the other parameters were set to default values. All the parameters are shown in Additional file 1.

### Identification accuracy

We evaluated the accuracy of the identifications reported by the ISC11 database search as well as the accuracy of the localization of mass shifts in these identifications. In the evaluation, spectral identifications reported by the K12 database search were used to find “correct identifications” for the ISC11 database search.

Below we describe the method for mapping a K12 identification to an ISC11 identification. Let *Q* be a query spectrum, and *F*_K12_ the best scoring proteoform identified from the K12 database (Fig. 2). The proteoform *F*_K12_ is called the *K12 proteoform* of *Q*. The unmodified protein segment of the proteoform *F*_K12_ is called the *K12 protein segment* of *Q*, denoted by *S*_K12_. The protein segment *S*_K12_ is a subsequence or the full sequence of the database protein sequence of *F*_K12_. Similarly, the proteoform identified from the ISC11 database is called the *ISC11 proteoform* of *Q* and denoted by *F*_ISC11_. The unmodified protein segment of the proteoform *F*_ISC11_ is called *the ISC11 protein segment* of *Q*, denoted by *S*_ISC11_. Because the K12 database contains protein sequences of the target species and the 1% FDR cutoff used in spectral identification is stringent, we assume that the K12 proteoform *F*_K12_ is correct. We use two steps to map the K12 proteoform to its homologous protein segment in the ISC11 database: (1) We employ BLAST-P (version 2.3.31) [19] to search *S*_K12_ against the ISC11 database to find the best ISC11 homologous sequence, denoted by *P*_ISC11_. The K12 protein segment *S*_K12_ may be aligned with only a subsequence of *P*_ISC11_. (2) The R function “pairwiseAlignment” in package “Biostrings” [20] is used to find the best global-local alignment between *S*_*K*12_ and *P*_ISC11_. The subsequence of *P*_ISC11_ in the alignment, denoted by $S_{\mathrm {ISC11}}^{'}$, is called the *homologous protein segment* of *Q*. The match between the query spectrum *Q* and the homologous protein segment $S_{\mathrm {ISC11}}^{'}$ is treated as the correct ISC11 identification of *Q*. We compare $S_{\mathrm {ISC11}}^{'}$ with the ISC11 protein segment *S*_ISC11_ to evaluate the accuracy of the ISC11 proteoform *F*_ISC11_. If *S*_ISC11_ and $S_{\mathrm {ISC11}}^{'}$ are from the same protein, we say the ISC11 database search correctly identifies the target protein. If *S*_ISC11_ and $S_{\mathrm {ISC11}}^{'}$ are from the same protein and their sequences are also the same, we say the ISC11 database search correctly identifies the protein segment.

In the analysis of the EC data, an *E*-value cutoff of 0.01 in BLAST-P was used to filter identifications. Other parameters in BLAST-P were set to default values. In the function “pairwiseAlignment”, the penalty for a gap extension was set to 4, the penalty for a gap opening was set to 10, and all the other parameters were set to default values.

### Accuracy of mass shift localization

When a PrSM reported from the ISC11 database search correctly identifies the protein segment, we further study the accuracy of its mass shift localization. There are two types of unknown mass shifts in reported matches between spectra and ISC11 proteoforms: (1) The K12 proteoform of the query spectrum does not have any unknown alternations, and the K12 protein segment is the same as the K12 proteoform. In this case, the unknown mass shift in the ISC11 proteoform is the sum of the mass shifts of all the substitutions, insertions and deletions between the K12 protein segment and the ISC11 protein segment. TopPIC reports a subsequence *S*_SHIFT_ of amino acids in the ISC11 protein segment as the possible positions of the mutations for the unknown mass shift. If *S*_SHIFT_ covers the positions of all the substitutions, insertions and deletions, we say the reported subsequence *S*_SHIFT_ is correct. (2) The K12 proteoform has an unknown mass shift compared with the unmodified K12 protein segment. In this case, the unknown mass shift in the ISC11 proteoform is the sum of the mass shifts of all the substitutions, insertions and deletions between the K12 protein segment and the ISC11 protein segment as well as the unknown mass shift in the K12 proteoform. If *S*_SHIFT_ covers the positions of all the substitutions, insertions and deletions as well as those of the mass shift, we say the reported subsequence *S*_SHIFT_ is correct.

## Results

### Similarity between the K12 and ISC11 databases

**Fig. 3:**
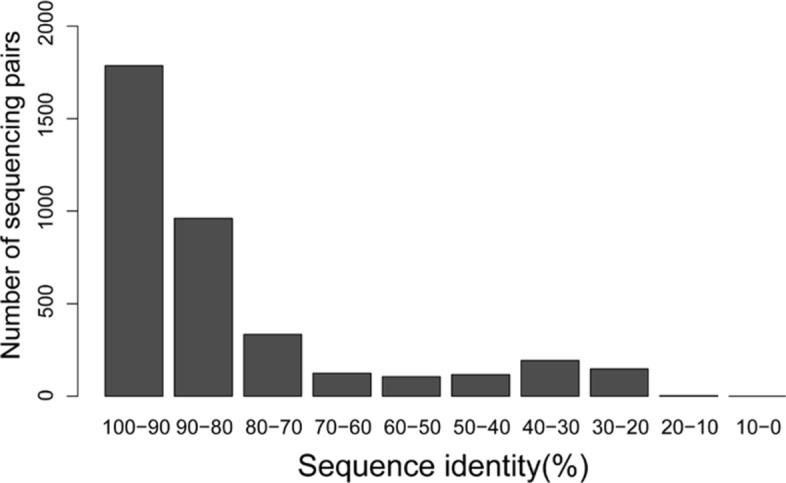
The histogram of the sequence identities between the 3769 protein sequences in the K12 proteome database and their corresponding homologous sequences reported by BLAST-P with a 0.01 *E*-value cutoff from the ISC11 proteome database

We analyzed the similarity between protein sequences in the K12 database and their homologous sequences in the ISC11 database. We used BLAST-P to search each protein sequence in the K12 database against the ISC11 database to find its homologous sequence with an *E*-value cutoff of 0.01. BLOSUM62 was used as the similarity matrix and other parameters were set to default values in BLAST-P. Of the 4312 K12 protein sequences, 3769 were aligned to homologous ISC11 protein sequences. BLAST-P reported a sequence identity (the percentage of identical matches over the entire length of the alignment) for each K12 and ISC11 protein sequence pair. The histogram of the reported sequence identities is given in Fig. 3. Of the 3769 sequence pairs, 1786 (47.4*%*) have an identity no less than 90%, and 960 (25.5*%*) have an identity between 80% and 90%. The sequence similarities are high for many reported homologous sequence pairs, making it possible to search the EC top-down MS data against the ISC11 database for proteoform identification.

### Comparison of identified proteoforms

**Fig. 4:**
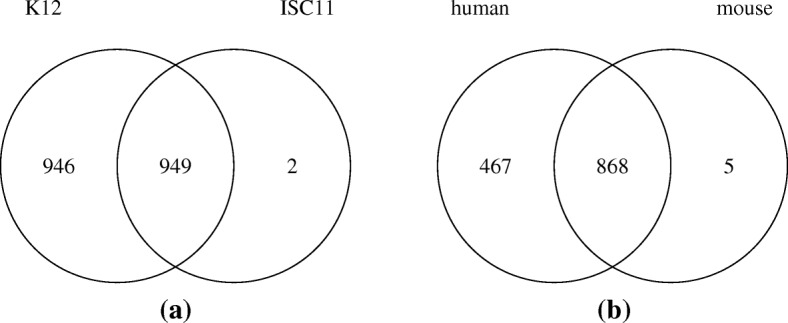
Comparison of the numbers of spectra identified by TopPIC with a 1% spectrum-level FDR using a proteome database of the target species and a homologous proteome database. (**a**) The EC data set; (**b**) the MCF-7 data set

A PrSM identified using the K12 database is called a K12 PrSM, and a PrSM identified using the ISC11 database is called an ISC11 PrSM. With a 1% spectrum-level FDR, TopPIC identified 1895 K12 PrSMs from 180 proteins and 951 ISC11 PrSMs from 98 proteins from the EC data set. A main reason for the low identification rate in the K12 database search is that many query spectra do not contain enough fragment masses for confident identification. Of the 4054 query spectra, 1323 contain no more than 30 fragment masses. The ISC11 database search identified 949 (50.1*%*) spectra and missed 946 spectra identified by the K12 database search (Fig. 4a and Additional file 2). With a relaxed 5% spectrum-level FDR, TopPIC identified 1062 ISC11 PrSMs, including 1058 of the 1895 spectra identified by the K12 database search with a 1% FDR. Most of the spectra identified by the ISC11 database search were also identified by the K12 database search, showing that most of the identified ISC11 PrSMs are accurate and that it is feasible to use a homologous database for spectral identification when no reference databases are available. The number of identified spectra with the ISC11 database is about half of that with the K12 database, demonstrating that using a homologous database significantly reduces the number of identifications.

For each of the 1895 identified K12 PrSMs, we obtained its K12 proteoform and K12 protein segment, and searched the K12 protein segment against the ISC11 database to find its homologous protein segment. (See Fig. 2 and “Methods” section.) Many ISC11 protein segments and homologous protein segments are truncated protein sequences, not whole ones. With an *E*-value cutoff of 0.01, BLAST-P identified an ISC11 protein segment for 1792 of the 1895 K12 PrSMs. For each of the 1792 PrSMs with a K12 protein segment and a homologous protein segment, the global alignment of the two segments was computed to obtain the number of mutations (substitutions, insertions, and deletions) between them. Of the 1792 segment pairs, a total of 1126 have no more than 4 mutations: 328 segment pairs are the same, 536 segment pairs contain only one or two mutations, and 262 segment pairs contain three or four mutations. When the homologous protein segment of a query spectrum contains 5 or more mutations, software tools for spectral identification often fail to identify it using homologous sequences. As a result, we focused on only the 1126 spectra with no more than 4 mutations in segment pairs, and these spectra are referred to as the high identity spectrum (HIS) set.

We also compared the performance of TopPIC on the MCF-7 data set with the human proteome database and the mouse proteome database. With a 1% spectrum-level FDR, TopPIC identified 1335 human PrSMs from 175 proteins and 873 mouse PrSMs from 112 proteins from the MCF-7 data set. The mouse database search identified about 868 (65.0*%*) of the spectra identified by the human database search. (Fig. 4b and Additional file 3).

### Identification rates using homologous sequences

Because unknown mass shifts were allowed in PrSMs reported by TopPIC, the 1126 K12 PrSMs of the HIS set were divided into two group: 569 without unknown mass shifts (perfect group) and 557 each with one unknown mass shift (mass shift group).

The ISC11 database search identified 447 (78.56*%*) of the 569 spectra in the perfect group and 375 (67.32*%*) of the 557 spectra in the mass shift group. In the perfect group, the ISC11 database search identified the correct protein for 444 spectra, and the correct protein segment for 438 spectra. (See “Methods” section.) In the mass shift group, the ISC11 database search identified the correct protein for 373 spectra and the correct protein segment for 361 spectra.

**Fig. 5:**
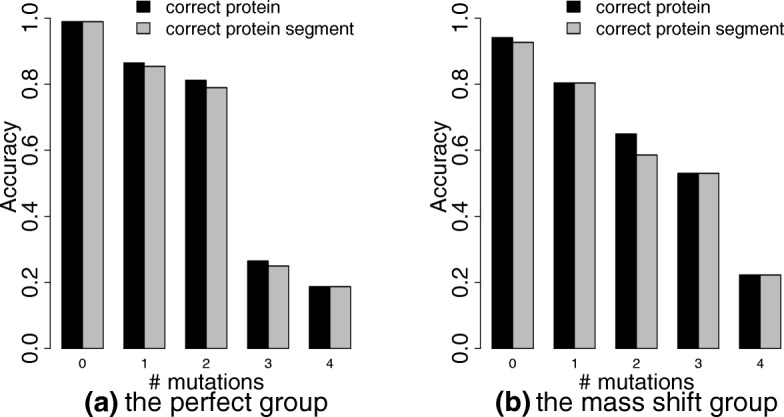
The CP and CS rates for the spectra in the 5 perfect subgroups *G*_0_, *G*_1_, *G*_2_, *G*_3_, *G*_4_ and in the 5 mass shift subgroups *H*_0_, *H*_1_, *H*_2_, *H*_3_, *H*_4_. (**a**) The perfect group; (**b**) the mass shift group

We investigated how the numbers of mutations in homologous sequences affect the sensitivity of spectral identification when the homologous ISC11 database was used. The 569 spectra in the perfect group were divided into 5 subgroups *G*_0_, *G*_1_, *G*_2_, *G*_3_, *G*_4_ (*G*_0_: 192 PrSMs, *G*_1_: 96 PrSMs, *G*_2_: 181 PrSMs, *G*_3_: 68 PrSMs, *G*_4_: 32 PrSMs) based on the numbers of mutations between their K12 segments and homologous protein segments. (See “Methods” section.) The subgroup *G*_*i*_ (0≤*i*≤4) contained the spectra corresponding to protein segment pairs with *i* mutations. The 557 spectra in the mass shift group were divided into 5 subgroups *H*_0_, *H*_1_, *H*_2_, *H*_3_, *H*_4_ (*H*_0_: 136 PrSMs, *H*_1_: 102 PrSMs, *H*_2_: 157 PrSMs, *H*_3_: 81 PrSMs, *H*_4_: 81 PrSMs) similarly. Let *n*_*t*_ be the number of spectra in a subgroup. Let *n*_*p*_ and *n*_*s*_ be the numbers of spectra in the subgroup with correct protein identifications and correct protein segment identifications in the ISC11 database search, respectively. We define the correct protein (CP) rate and the correct segment (CS) rate as the ratios $\frac {n_{p}}{n_{t}}$ and $\frac {n_{s}}{n_{t}}$, respectively. The CP and CS rates for the 10 subgroups are shown in Fig. 5.

As the number of mutations per protein segment pair increases from 0 to 4, the CS rate for the perfect group drops from 99.0*%* to 18.8*%*, and that for the mass shift group drops from 92.7*%* to 22.2*%*. The CS rates are above 79% for *G*_1_, *G*_2_, and *H*_1_, showing that a good sensitivity can be obtained when homologous sequences have high similarity (no more than 2 mutations). The CS rates are below 25% for *G*_3_ and *G*_4_. In general, the mass shift subgroups have lower CS rates than the perfect subgroups because of the existence of unknown mass shifts in identified proteoforms. In the 10 subgroups, the CS rates are similar to the CP rates, demonstrating that the ISC11 database search can identify the correct protein segment in most cases when the correct protein is identified.

### Accuracy of mass shift localization

The localization of mass shifts is an important step in interpretation of top-down mass spectra. We chose only PrSMs with correct protein segment identifications from *G*_1_,…,*G*_4_,*H*_1_,…,*H*_4_ for the evaluation of mass shift localization. A total of 248 PrSMs were selected from the perfect group, and 235 from the mass shift group. The mass shifts were correctly localized in 132 (53.2*%*) of the 248 PrSMs in the perfect group and in 35 (14.9*%*) of the 235 PrSMs in the mass shift group (See “Methods” section.) The accuracy of mass shift localization of the perfect group is much higher than the mass shift group. The main reason is that PrSMs in the mass shift group contains unknown alterations as well as mutations, making it challenging to accurately localize mass shifts. Many errors in mass shift localization are due to randomly matched fragment peaks in identified PrSMs.

## Discussion

Most existing software tools [13, 14] are capable of identifying proteoforms with variable PTMs, but not proteoforms with unknown mutations. In homologous sequence comparison, there are in total 20×19=380 possible types of mutations: each of the 20 amino acids can be mutated to one of the other 19 amino acids. It is impractical to treat all the 380 mutations as variable PTMs in spectral identification. In general, these software tools are not capable of identifying proteoforms with unknown mutations when homologous sequences are used in database search. TopPIC and the delta-M mode of ProSightPC [8] are capable of identifying proteoforms with unknown alterations and can be used in top-down MS data analysis when the species being studied lacks a complete proteome database.

## Conclusions

In this paper, we studied the proteoform identification problem by database search using top-down MS and evaluated the performance of TopPIC on two data sets with homologous sequences. The experimental results show that mutations in homologous sequences are a crucial factor that affects the sensitivity and accuracy of proteoform identification. While the sensitivity of TopPIC dropped significantly in spectral identification with homologous sequences with 3 or more mutation, it achieved a high sensitivity and accuracy with homologous sequences with 1 or 2 mutations. The results demonstrate that it possible to use homologous protein databases in top-down spectral identification.

## Additional files

Additional file 1 Supplementary material. (PDF 61 kb)

Additional file 2 Identifications from the EC data set. (XLSX 583 kb)

Additional file 3 Identifications from the MCF-7 data set. (XLSX 351 kb)

## Abbreviations

CID: Collision-induced dissociation
CP: Correct protein
CS: Correct segment
Da: Dalton
EC: Escherichia coli
ETD: Electron-transfer dissociation
FDR: False discovery rate
HIS: High identity spectrum
MS: Mass spectrometry
MS/MS: Tandem mass spectrometry
ppm: Parts-per million
PrSM: Proteoform spectrum match
PTM: Post-translational modification
RPLC: Reverse phase liquid chromatography
