# Supplementary material for: Evaluation of top-down mass spectral identification with homologous protein sequences
Source: BMC Bioinformatics. 2018 Dec 28;19(Suppl 17):494. doi: 10.1186/s12859-018-2462-1 (PMC6309053; doi:10.1186/s12859-018-2462-1)
Supplement: Supplementary file 1 — Supplementary material. (PDF 61 kb) [file 12859_2018_2462_MOESM1_ESM.pdf]

Evaluation of top-down mass spectral identification with homologous protein sequences(Supplementary material)

1 Parameter settings of TopPIC

Table 1: Parameter settings of TopPIC

**Parameter**	**Value**
Fragmentation method	FILE
Fixed modifications	None
N-terminal forms of proteins	NONE, NME, NME+ACETYLATION
Using a decoy database	Yes
Error tolerance	15 ppm
Maximum number of unexpected modifications (unknown mass shifts) in a PrSM	0
Cutoff type	FDR
Cutoff value	0.01/0.05
Using the generating function approach to compute p-values and E-values	No
Number of combined spectra	1
Common modifications for characterization of unknown mass shifts	None
E-value computation	Lookup table
